# Effectiveness of health education in the self-care and adherence of patients with heart failure: a meta-analysis

**DOI:** 10.1590/1518.8345.4281.3389

**Published:** 2021-07-19

**Authors:** Juliana de Melo Vellozo Pereira Tinoco, Lyvia da Silva Figueiredo, Paula Vanessa Peclat Flores, Bruna Lins Rocha de Padua, Evandro Tinoco Mesquita, Ana Carla Dantas Cavalcanti

**Affiliations:** 1 Universidade Federal Fluminense, Hospital Universitário Antônio Pedro, Niterói, RJ, Brazil.; 2 Universidade Federal Fluminense, Niterói, RJ, Brazil.; 3 Scholarship holder at the Coordenação de Aperfeiçoamento de Pessoal de Nível Superior (CAPES), Brazil.; 4 Universidade Federal Fluminense, Escola de Enfermagem Aurora de Afonso Costa, Niterói, RJ, Brazil.

**Keywords:** Self Care, Heart Failure, Patient Complicance, Health Education, Health Outcome, Treatment Adherence and Compliance, Autocuidado, Insuficiência Cardíaca, Cooperação do Paciente, Educação em Saúde, Resultados de Saúde, Cooperação e Adesão ao Tratamento, Autocuidado, Insuficiencia Cardíaca, Cooperación del Paciente, Educacion em Salud, Resultado del Tratamiento, Cumplimiento y Adherencia al Tratamento

## Abstract

**Objective:**

to evaluate in the literature the effectiveness of the health education interventions in self-care and adherence to treatment of patients with Chronic Heart Failure.

**Method:**

a systematic review with meta-analysis. Studies were selected that compared health education interventions with the usual care to assess the outcomes of adherence and self-care. The quality of the methodological evidence was assessed by the Grading of Recommendations, Assessment, Development and Evaluation system.

**Results:**

the educational interventions were more effective in relation to the usual care in the outcome of adherence (fixed effect=0-3841; p-value <0.001). There was no statistical difference in the outcome of self-care (fixed effect=0.0063; p-value=0.898).

**Conclusion:**

the educational interventions improved the outcome of adherence, though not self-care in the patient with Heart Failure.

## Abstract (pt)

**Objetivo:**

avaliar na literatura a efetividade das intervenções de educação em saúde na adesão e autocuidado ao tratamento de pacientes com Insuficiência Cardíaca Crônica.

**Método:**

revisão sistemática com metanálise. Foram selecionados estudos que comparavam intervenções de educação em saúde com o cuidado usual para avaliar os desfechos de adesão e autocuidado. A qualidade da evidência metodológica foi avaliada pelo sistema *Grading of Recommendations, Assessment, Development and Evaluation*.

**Resultados:**

as intervenções educativas foram mais efetivas em relação ao cuidado usual no desfecho de adesão (efeito fixo =0,3841; p-valor <0,001). Não houve diferença estatística no desfecho de autocuidado (efeito fixo =0,0063; p-valor =0,898).

**Conclusão:**

as intervenções educativas melhoraram o desfecho de adesão, mas não o de autocuidado no paciente com Insuficiência Cardíaca.

## Abstract (es)

**Objetivo:**

evaluar en la literatura la efectividad de las intervenciones de educación en salud para la adhesión al tratamiento y el autocuidado de pacientes con Insuficiencia Cardíaca Crónica.

**Método:**

revisión sistemática con meta-análisis. Se seleccionaron estudios que comparaban intervenciones de educación en salud con el cuidado habitual para evaluar los resultados de adhesión al tratamiento y autocuidado. La calidad de la evidencia metodológica se evaluó con el sistema *Grading of Recommendations, Assesment, Development and Evaluation*.

**Resultados:**

las intervenciones educativas fueron más efectivas en relación al cuidado habitual en el resultado de adhesión al tratamiento (efecto fijo=0,3841; valor p <0,001). No se registró diferencia estadística en el resultado de autocuidado (efecto fijo=0,0063; valor p =0,898).

**Conclusión:**

las intervenciones educativas mejoraron el resultado de adhesión al tratamiento, aunque no el de autocuidado en el paciente con Insuficiencia Cardíaca.

## Introduction

Heart Failure (HF) is a complex clinical syndrome, in which dyspnea, fatigue and fluid retention can limit tolerance to exercise and functional capacity^1^. Despite advances in pharmacological and non-pharmacological follow-up, HF affects thousands of people worldwide and is associated with frequent use of the health services^2^.

A number of studies point out that the prevalence of HF can affect nearly 1%-2% of the world population, with 6% to 10% of the individuals being over 65 years old^3^^-^ 4. In Brazil, between June 2018 and June 2019, HF totaled 212,208 thousand cases of hospitalizations and 24,035 thousand deaths. These numbers point to HF as the leading cause of hospitalization for diseases of the circulatory system and the second leading cause of mortality in Brazil^5^.

Lack of adherence to the therapeutic regimen, especially with regard to lifestyle changes, is one of the factors that contribute for decompensation episodes and readmission due to HF^1^^,^ 6 ^-^ 7. However, one of the main reasons for lack of adherence is the low capacity of the individuals to exercise their self-care^8^^-^ 9, which is understood as the natural decision-making process of individuals and their families, aimed at both the prevention and treatment of the disease^10^^-^ 11.

In HF, the capacity for self-care can be limited by low health literacy, cognitive deficit, depressive symptoms, presence of multiple comorbidities and low self-efficacy to perform self-care^12^^-^ 14.

The low health literacy in patients with HF translates into less knowledge related to the disease, worse self-care behavior, low quality of life and decreased medication adherence prescribed in HF. It is also associated with the incidence of mortality in outpatients and inpatients. In addition, it is emphasized that low health education can predict morbidity and mortality^15^^-^ 16.

These factors directly affect patient adherence and interaction with health professionals, since it is a complex process that ranges from complying with and following the treatment prescribed in search for well-being and health, represented by changes in the lifestyle that include attending appointments and greater control of the medication^17^.

A number of studies indicate that, with an adequate guidance on the disease and patient involvement in self-care, health outcomes improve^18^^-^ 19. Patient education is a fundamental component of HF care management programs, in addition to optimizing clinical treatment and psychosocial support. This strategy reduces hospitalization due to HF and mortality in post-discharge patients^20^.

There are currently several educational interventions for patients with HF in order to improve self-care and adherence to the treatment. However, the researchers did not find in the literature any synthesis of evidence on educational interventions and the impact of these interventions on self-care and adherence, in order to generate recommendations for clinical decision-making in the professional practice. Similar articles were assessed in order to reduce subjectivity by standardizing measures of effect, in addition to making recommendations on which interventions are most effective for the outcomes proposed in this study.

This review can guide health professionals who work in clinics specialized in HF, outpatient clinics or in the hospital environment when making decisions about the best educational intervention to achieve self-care and adherence with HF patients.

Thus, this study aimed to assess in the literature the effectiveness of the health education interventions in self-care and adherence to the treatment of patients with Chronic Heart Failure.

## Method

This is a systematic review with meta-analysis, conducted according to the precepts of the Joanna Briggs Institute (JBI) - Evidence Synthesis Groups, in addition to the indications of the Preferred Reporting Items for Systematic Reviews and Meta-Analyses (PRISMA)^21^.

The protocol entitled “The effectiveness of interventions in health education in the adherence to treatment and self-care of patients with heart failure: a systematic review” is published in the PROSPERO platform under number CRD42018094051.

The review had the following guiding question: What is the effectiveness of health education in the adherence to the treatment and self-care of patients with HF?

For the elaboration of the guiding question and search for articles, the PICO strategy was used, which is an acronym for Patient/Problem (heart failure), Intervention (health education), Control/Comparison and Outcomes (patient compliance, self-care)^22^. It should be noted that, in the PICO strategy of this study, the “C” was not inserted as a specific intervention, since any intervention deemed as control in the articles was considered for comparison analysis with health education interventions.

The following inclusion criteria were adopted: studies with adults over 18 years of age with HF, addressing a health education intervention for adherence to the treatment and/or self-care; indexed in databases published in English, Spanish or Portuguese between 2012 and 2019, regardless of the professional area. The time cut is justified by the need to check health education interventions updated in light of the technological and health advances in recent decades.

This review considered studies with an experimental or quasi-experimental design, such as without randomization with a single group pre- and post-test, observational, including prospective and retrospective cohort, case-control and cross-sectional studies.

Since there are different methods for assessing adherence and self-care, as a way to standardize the analysis with the best possible scientific evidence, the studies considered were those that presented an evaluation of the referred outcomes through questionnaires with validated psychometric assessments, with the possibility of generating a final score, as a way of comparing the studies found.

Due to the statistical tests performed for this meta-analysis, this research considered studies with only two intervention groups. Studies that did not have a clear methodology and did not answer the study question were excluded.

The search was carried out in the following databases: PubMed, Cumulative Index of Nursing and Allied Health (CINAHL), LILACS, Cochrane and Scopus. The search for articles began in September 2019, through the registration on the website of the Federated Academic Community (Comunidade Acadêmica Federada, CAPES CAFe). The controlled descriptors were obtained through the Health Sciences Descriptors (Descritores em Ciências da Saúde, DeCS), MeSH (Medical Subject Headings) terms and CINAHL headings, according to the specificity of each database.

The descriptors selected were the following: heart failure/insuficiência cardíaca, health education/educação em saúde, patient compliance/cooperação do paciente, self-care/autocuidado, which were used for all search in the databases identically, using the Boolean operator “and” in the searches.

To achieve better results, the search was divided into two moments, one with the “patient compliance” descriptor and the other with the “self-care” descriptor. This division occurred because, by inserting patient compliance or self-care, the search was nonspecific, that is, there were many studies that were not related to the study objective. In this sense, according to what is recommended by PRISMA, the search strategy carried out in the PubMed database follows below, for future repetition:

For the patient compliance descriptor: ((“heart failure”[MeSH Terms] OR (“heart”[All Fields] AND “failure”[All Fields]) OR “heart failure”[All Fields]) AND (“health education”[MeSH Terms] OR (“health”[All Fields] AND “education”[All Fields]) OR “health education”[All Fields])) AND (“patient compliance”[MeSH Terms] OR (“patient”[All Fields] AND “compliance”[All Fields]) OR “patient compliance”[All Fields]).

For the self-care descriptor: ((“heart failure”[MeSH Terms] OR (“heart”[All Fields] AND “failure”[All Fields]) OR “heart failure”[All Fields]) AND (“health education”[MeSH Terms] OR (“health”[All Fields] AND “education”[All Fields]) OR “health education”[All Fields])) AND (“self-care”[MeSH Terms] OR (“self”[All Fields] AND “care”[All Fields]) OR “self-care”[All Fields])

The search in the databases was carried out by the main researcher, who subsequently forwarded the abstracts of the articles found to two reviewers, who carried out the assessment blindly. The articles were assessed regarding titles and abstracts, where the eligibility criteria were applied; a third reviewer, who is trained by the JBI, assessed the possible divergences that occurred in the selection of abstracts for the final decision on whether to include or exclude them.

The abstracts assessed returned to the main researcher, who then made all articles available in full-text format to the reviewers, who methodologically and blindly assessed them again. For this, the instruments of the Joanna Briggs Institute were used. At the end of this process, the articles that reached 70% of utilization were selected^23^. Possible disagreements or doubts about the methodological assessment of the articles were clarified by a third reviewer trained by the JBI.

To reduce the possible risk of bias in the selection of studies, refinement was performed independently by two evaluators looking for 100% agreement; in addition, the same rule was applied to the results of the selection of studies, and there should be no divergence in the number of studies selected in each database.

In addition, the reviewers received from the qualified reviewer of the JBI a theoretical and practical systematic review training with a two-hour workload per instrument, totaling eight hours. This instrument consists of questions that assess the methodological quality of each review study included in the SR according to the method used.

The quality of the methodological evidence of the studies and the strength of recommendation were analyzed according to the precepts of the GRADE (Grading of Recommendations, Assessment, Development and Evaluation) system. This assessment considered the study design, its execution, consistent results, evidence, limitations, and data scarcity, as well as the probability of bias. The GRADE system considers four levels of evidence. There is high (A) evidence when other studies are unlikely to change the estimated confidence in the effect of the intervention. There is moderate (B) evidence when there is moderate confidence in the estimated effect. When confidence in the effect is limited, it is considered low (C) and, when any effect estimate is uncertain, there is a very low level (D)^24^.

The data were organized in charts and tables for the synthesis of information from the journals, such as: title, author’s name, year of publication, in addition to the sample size, outcome result (mean) and standard deviation (or variance) of the control and intervention groups.

The means, standard deviation and sample size of the study were calculated. To calculate the effect size, the difference of means was performed, divided by the combined standard deviation, multiplied by a correction factor. To calculate the weights in each study, the inverse variance method was applied^25^.

The Fixed Effect and Random Effect models were used to represent the data and estimate the overall effect of the study. The fixed effect is a form of modeling that treats the selected studies as unique, thus the degree of importance (or weight) for calculating the overall effect is different for each study. The weight size is influenced by the quality of the estimates presented by the study. Two factors are decisive for calculating the weight, the variability found and the sample size used^25^.

The results of the meta-analysis were presented using the forest plot graph, where each study is shown with its respective effect size, 95% confidence interval, the weights for each model (fixed and random) and the estimate of the overall effect of the study^25^. In order to account for the effect size, the standardized mean difference was used for both scales. The results were obtained by using the meta package of the R software.

## Results

**Figure 1:**
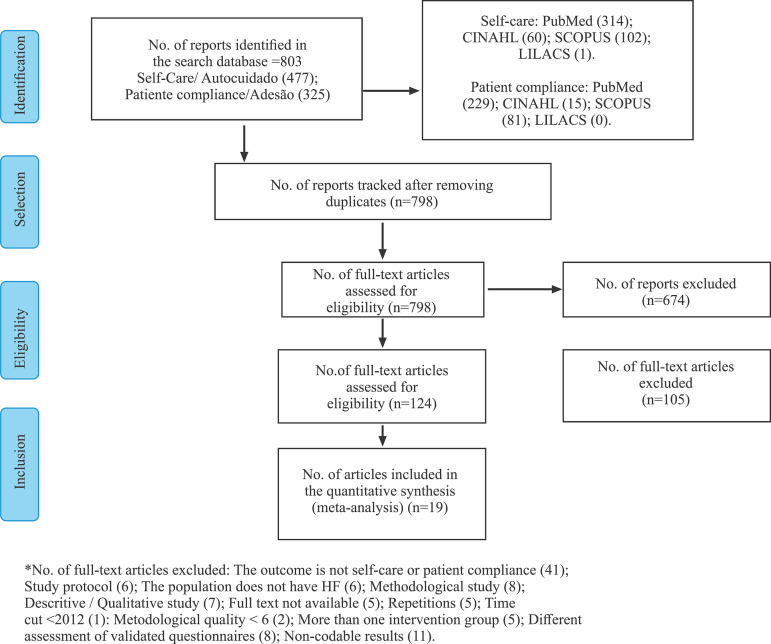
*PRISMA* flow diagram of selection of the studies. Niterói, RJ, Brazil, 2019

The search resulted in 802 studies which, after applying the eligibility criteria, derived in 19 studies: 15 with self-care outcome, and five (5) with adherence outcome. The study inclusion process is described in Figure 1.

Figure 2 briefly presents the respective studies included, with health education strategies presented to the patients randomized to the intervention group, with the various questionnaires validated to assess the self-care and adherence outcomes. The assessment scores in relation to the methodological quality of the JBI demonstrate that the articles obtained more than 70% of utilization.

**Figure 2:** Articles included for meta-analysis. Niterói, RJ, Brazil, 2019

Study/ Country/ Year	Intervention/Professional	Type of study	Self-care Scales		Adherence Scale				JBI score**
			EHFSCBC*	SCHFI^†^	MARC^‡^	MMAC^§^	MOSSAS-3HF^||^	QA-26^¶^	
Study 1(26) USA, 2012	Cognitive training. Nurse	RCT^††^		X					12
Study 2(27) Netherlands, 2013	Nursing consultation + Telemonitoring. Nurse	RCT^††^	X						12
Study 3(28) USA, 2017	Education for self-care/cultural adaptations + Phone consultation. Nurse	RCT^††^		X					12
Study 4(29) USA, 2015	Home Visit + Phone consultation by motivational interview. Nurse	RCT^††^		X					12
Study 5(30) China, 2015	Transitional care. Nurse	RCT^††^		X					11
Study 6(31) Australia, 2015	Electronic resource/Cultural adaptation. Indigenous researcher	QE^‡‡^		X					07
Study 7(32) USA, 2014	Group guidance + Lifestyle coaching. Health educator	RCT^††^		X					11
Study 8(33) Iran, 2013	Education for self-care after discharge + Phone consultation. Nurse	RCT^††^		X					13
Study 9(34) Iran, 2017	Education for self-care by Motivational Interview. Nurse	RCT^††^		X					11
Study 10(35) USA, 2015	Cognitive Behavioral Therapy	RCT^††^		X					11
Study 11(36) Korea, 2018	Nursing consultation + Phone consultation. Nurse	QE^‡‡^	X						09
Study 12(37) Iran, 2015	Support group for patients and family members. Nurse	QE^‡‡^	X						12
Study 13(38) Netherlands, 2014	Telemonitoring system (Health Buddy^®^) + Usual care. Nurse	RCT^††^	X						12
Study 14(39) Brazil, 2013	Home visit after hospital discharge + Phone consultation. Nurse	RCT^††^	X					X	12
Study 15(40) USA, 2015	Education for self-care + Phone consultation + Usual care. Nurse	RCT^††^	X						13
Study 16(41) Germany, 2016	Self-managed educational group. Multi-professional team (physician, nurse, psychologist, and physiotherapist)	RCT^††^			X				11
Study 17(42) China, 2015	Health education booklet + Phone consultation. Physician and nurses	RCT^††^				X			11
Study 18(43) USA, 2018	Guidance through ultrasound of the inferior vena cava. Nurse	RCT^††^					X		11
Study 19(44) China, 2014	Home visit + Phone consultation. Nurse	RCT_††_						X	12

* EHFSCBC = European Heart Failure Self-care Behaviour Scale;

† SCHFI = Self-Care of Heart Failure Index;

‡ MARC = Medication Adherence Report Scale;

§ MMAC = Morisky Medication Adherence Scale;

|| MOSSAS-3HF = Medical Outcomes Study Specific Adherence Scale modified for HF;

¶ QA-26 = 26-point adherence questionnaire;

** JBI = Joanna Briggs Institute;

†† RCT = Randomized Clinical Trial;

‡‡ QE = Quasi-experimental

The studies evaluated self-care in a total of 1,841 HF patients. For the outcome of adherence, a total of 974 individuals participated in the analyzed studies. These studies are mostly from the United States and China, from 2012 to 2018, with the majority published in 2015. Of these, 11 studies used combined strategies.

It should be noted that, in the studies analyzed, the control group was treated with usual care, which was described in the studies as routine outpatient follow-up pursuant to institutional protocols.

Table 1 shows the size of the global effect for the self-care and adherence outcomes, respectively, according to the fixed and random models. With respect to self-care, the fixed effect was not significant; thus, the control and intervention groups produced similar results. The global effects obtained by applying the fixed and random effects models were 0.0063 and 0.6799, respectively. The models did not have convergent results. The fixed-effect model showed a non-significant result (p-value=0.8986), showing that there are no differences in the self-care scale between the groups. However, the random-effect model obtained a significant result (p-value=0.0091) in favor of the effect in the experimental group. Therefore, on average, the self-care results in the experimental group were higher than in the control group.

**Table 1:** Result of the global effect size for the self-care and adherence scale, by model. Niterói, RJ, Brazil, 2019

Model	Estimate	95% confidence interval	z-value	p-value
Outcome: Self-care				
Fixed	0.0063	(-0.0903; 0.1028)	0.13	0.8986
Random	0.6799	(0.1690; 1.1907)	2.61	0.0091
Outcome: Adherence				
Fixed	0.3841	(0.2533; 0.5147)	5.76	< 0.001
Random	0.7604	(0.0038; 1.5170)	1.97	0.0489

Regarding the adherence outcome, the global effects obtained by applying the fixed and random effects models were 0.3841 and 0.7604, respectively. The models had convergent results. Both models were significant (fixed p-value <0.001 and random p-value 0.048). Therefore, on average, the results of adherence in the experimental group were higher than in the control group.

Table 1 - Result of the global effect size for the self-care and adherence scale, by model. Niterói, RJ, Brazil, 2019

**Figure 3:**
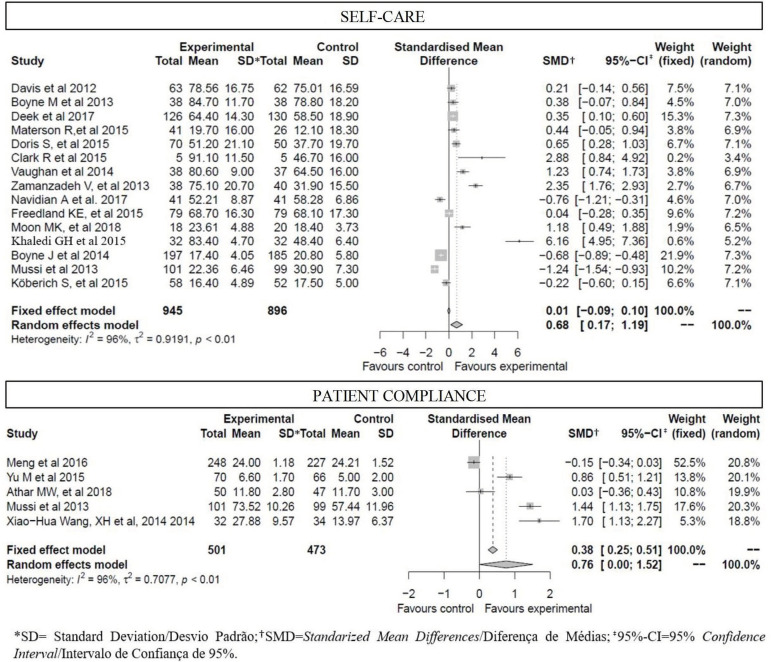
Result of applying the meta-analysis for the self-care and adherence outcomes, considering both fixed- and random-effect models. Niterói, RJ, Brazil, 2019

Figure 3 presents the results of the meta-analysis considering the self-care and adherence outcomes, respectively. It was verified that, for self-care, studies No. 6 and No. 12 were those that came closest to the results in favor of the experimental group (intervention)^31^^,^ 37.

For the adherence outcome, the results were statistically significant in favor of the experimental group due to the fixed effect. Despite the small number of studies, the weights are distributed in an almost similar way among the papers.

The interventions implemented in these studies were, for the most part, two combined strategies: home visit (HV) + Phone consultation (PC) or PC with application of an educational leaflet^39^^-^ 40 ^,^ 42 ^,^ 44. There were also two individual strategies (personalized guidance based on the inferior vena cava ultrasound examination and educational group)^41^^,^ 43. Two studies (No. 14 and No. 19) that used HV + PC were the ones that came closest to the result in favor of the experimental group for the adherence outcome^39^^,^ 44.

In Figure 4, the quality of evidence of the results assessed by the GRADE system was considered low for both outcomes, which suggests that confidence in the effect is limited. The risk of bias, inconsistency and imprecision were the main factors that determined the low quality of evidence in the studies assessed.

**Figure 4:** GRADE Assessment. Niterói, RJ, Brazil, 2019

No. of the study	Study design	Risk of bias*	Inconsistency^†^	Uncertainty if the evidence is direct	Imprecision^‡^	Publication bias	Quality	Importance
Outcome: Self-care (Follow-up: 30 to 365 days, assessed by the following scales: EHFSCBC^§^ and SCHIFI^||^)								
15	Randomized Clinical Trial	Important	Important	Not important	Important	No	Low	Important
Outcome: Adherence (Follow-up: 30 to 180 days, assessed by the following scales: MARC^¶^, MMAC**, MOSSAS-3HF^††^ and QA-26^‡‡^ )								
5	Randomized Clinical Trial	Not important	Important	Not important	Important	No	Low	Important

* Risk of bias = A quasi-experimental study with no allocation between control/intervention;

† Inconsistency = 96% heterogeneity;

‡ Imprecision = There are initial studies and with a small number of events;

§ EHFSCBC = European Heart Failure Self-care Behaviour Scale;

|| SCHFI = Self-Care of Heart Failure Index;

¶ MARC = Medication Adherence Report Scale;

** MMAC = Morisky Medication Adherence Scale;

†† MOSSAS-3HF = Medical Outcomes Study Specific Adherence Scale modified for HF;

‡‡ QA-26 = 26-point adherence questionnaire

## Discussion

The meta-analysis demonstrated that there was an improvement in the outcome of adherence to the treatment in the patients from the experimental group, both in the fixed-effect and in the random-effect models. The self-care outcome did not differ between the experimental and control groups when assessing the fixed model.

The interventions presented in the studies with effect on adherence to the treatment were predominantly combined strategies between HV+PC or PC with application of educational material. The individual strategies were the educational group in HF and health guidance based on the ultrasound of the inferior vena cava. From these studies, it was evidenced that those who used HV+PC were the ones that had better significant results in favor of the experimental group^39^^,^ 44.

Corroborating this finding, in an experimental study with 201 patients, telephone follow-up took place seven and 30 days after discharge. There was an improvement in the outcome of medication and non-medication adherence after 90 days in the intervention group in relation to the control group (p<0.001)^45^.

In a before-and-after experimental study conducted with patients hospitalized due to decompensated HF, in two reference institutions in Rio Grande do Sul, the intervention consisted of three home visits after hospital discharge, with an interval of 45 days. The outcome of adherence to the treatment was assessed using a validated questionnaire, in the first and third HVs. 32 patients were included, with a mean age of 65±16 years old. The adherence scores were 16±2.6 vs. 20.4±2.7 on the first and third visits (p=0.001). After the intervention, an increase in the score of adherence to the treatment of the patients with HF was evidenced, highlighting the improvement in questions related to daily weight verification and restriction of water intake^46^.

Personalized medicine is a current and extremely relevant approach, since it considers the particularities of each patient^47^. One of the studies included in this review used ultrasound images (USG) as an education strategy for patients with decompensated acute heart failure, relating the ultrasound image of their inferior vena cava (IVC) with its liquid state (congestion)^43^. Although this study did not show positive effects, another showed that an intervention that included educational materials based on images reduced hospitalization or death for a period of 12 months^48^.

It has been shown that the educational group is a beneficial strategy in adherence to the treatment. A randomized clinical trial conducted in Brazil tested the effect of the guidance group on adherence to the treatment and self-care in patients with heart failure, showing an improvement from 13.9±3.6 to 14.8±2.3, from the initial to the final scores^14^. The authors concluded that, even with little difference in the initial and final scores, the adoption of an educational program is an important strategy in the health sector, especially in patients with chronic diseases^14^; however, it cannot guarantee a change in behavior because the relationship between what people know and adopt as a life habit is a tenuous and individual thing, which can be affected by several variables.

With regard to self-care, the fixed effect was not significant; thus, the control and experimental groups produced similar results. It is observed that three papers concentrate almost 40% of the global fixed effect, since they are studies with a good sample size and little variability found^38^^-^ 40. Therefore, in the fixed-effect model, the papers differ in the degree of importance. Thus, it is not possible to state that the experimental effect produces differentiated results compared to the control group only when considering the random effect, in which the studies are treated as a sample of studies on the theme.

A systematic review identified 14 instruments for measuring self-care in patients with HF and two of them had undergone rigorous psychometric tests: European Heart Failure Self-Care Behaviour Scale (EHFScBS) and Self-Care of Heart Failure Index (SCHFI)^49^. Other instruments are discussed in the literature, such as the Self-Care Behaviors Questionnaire (SCBQ)^50^ and the Test of Functional Health Literacy in Adults (S-TOFHLA), which assesses the level of health literacy, with psychometric validation for Brazil^51^^-^ 52.

Although the fixed effect did not show differences between the experimental and control groups, the studies that individually came closest to the result in favor of the experimental group were related to the electronic resource^31^ and to the support group for patients and family members^37^.

The complexity of self-care in HF can pose threats to the proposal and adherence to treatments, especially in patients with low health literacy. This, in turn, is associated with the inability to process, understand and put into practice information about the disease, resulting in difficulties to understand and follow the guidelines for the proposed treatment, resulting in greater morbidity and mortality^53^^-^ 54. Recent studies have already associated low HF literacy with a deficit in knowledge of the disease, low self-care, readmissions and mortality^54^^-^ 55.

Socioeconomic, demographic and educational factors are determining factors in low health literacy. Patients with these conditions generally have difficulty processing information about the treatment of the disease, such as reading notes and labels on medications, and understanding verbal information from their health professionals and educational materials^16^.

Patients with HF receive a range of health information and are often approached as passive recipients by health professionals, with little interaction between them. On the other hand, responsibility for self-care is often required. Therefore, it is of utmost importance to recognize the factors that interfere in the patient’s understanding and participation in the management of the disease and treatment, so that they are analyzed in view of the implementation of strategies in order that the interventions occur more effectively and satisfactorily.

The national and international HF Guidelines indicate HF treatment programs as Class I, level of evidence “A” for improving adherence, self-care and quality of life, as well as reducing hospitalizations, mortality and hospital costs. The main component of this program is its multi-professional constitution, focused on the education of patients and caregivers, whether on an outpatient or inpatient level, when planning discharge, using strategies such as face-to-face consultations, delivery of educational materials, telemonitoring and follow-up^1^^,^ 20.

Although the quality of the evidence of the results assessed by the GRADE system was considered low, this meta-analysis showed that combined educational strategies applied in patients with HF improved the compliance of patients with HF. Among them, the home visit together with phone consultation (HV+PC) is highlighted.

From the perspective of the work of nurses who handle HF patients, whether in HF outpatient clinics or during hospitalization, telephone consultation is considered a low-cost, easy-to-apply and quick-access strategy for patients. Home visits have the advantages of evaluating in loco and in real time the current situation of the patient, with the possibility of immediate interventions. The main disadvantage is the difficulty in accessing homes in at-risk crowded areas, both due to local violence and climatic conditions (landslides, flooding) and to the dependence on transportation for the health team.

In 2019, the American guideline for hospitalized patients with HF recommended that the PC should be performed with the patient and/or caregiver between 48 and 72 hours after discharge in order to check for signs of congestion, adherence to the treatment, clarification of doubts not discussed during hospitalization, and adequate access to prescription drugs^56^. The HV must be performed as soon as possible after discharge to reassess the clinical status and risk factors for readmission^56^.

Therefore, considering the findings of this study, the researchers recommend, as a priority, for better adherence to the treatment of HF patients, the adoption of combined HV+PC strategies.

The reality of the HF patients treated by the Unified Health System (Sistema Único de Saúde, SUS) denotes characteristics of high vulnerability for recurrent hospitalizations^57^. Considering this aspect, telephone consultations combined with home visits, implemented in family health modules and specialized clinics, promote the following benefits: reduced intervals between face-to-face consultations; increased bond and trust between patients, family members and health professionals; situational diagnosis of the patient and family and prevention of hospitalizations due to HF decompensation for preventable causes, decreasing hospitalization costs by the SUS^58^.

It is suggested that public institutions of basic/specialized care add the HV+PC to their respective local care flows in referral and counter-referral systems in the access to patients with HF treated by the SUS.

In this meta-analysis, the method of assessing adherence occurred by applying validated questionnaires as a way to standardize the analysis. However, this outcome can be assessed in several ways, such as the number of medications taken per patient per week/month, the measurement of serum biomarkers, and clinical examination. Given the diversity of estimates of this outcome, the results in this study must be analyzed with caution.

Like the self-care outcome, the studies that measured adherence involved very different samples. In addition, the non-conformity of studies regarding the presentation of results with measures of mean and standard deviation or median made it impossible to include other studies that could have been contemplated in the meta-analysis, which is a limitation of this study.

This study highlights the limitations in conducting the meta-analysis related to the different ways of measuring self-care adherence today, as well as the high sample variability of the included studies. The methodology used in the statistical tests allowed for the analysis of studies with only two intervention groups. Further analyses are necessary to better estimate strategies that effectively impact on self-care in patients with HF.

## Conclusion

Educational interventions improved the outcome of adherence, but not that of self-care in patients with HF. More detailed analyses are necessary, with the association of other clinical outcomes in order to consolidate the effect of the educational interventions on this population. It is suggested to include in future studies the outcome of health literacy to deepen the understanding and optimization of health education actions in HF.
